# Diabetic retinopathy is associated with diastolic dysfunction in type 2 diabetic patients with non-ischemic dilated cardiomyopathy

**DOI:** 10.1186/s12933-017-0566-y

**Published:** 2017-07-06

**Authors:** Yoo-Ri Chung, Se-Jun Park, Ka Young Moon, Seoyoung Annie Choi, Hong-Seok Lim, Sung Wook Park, Jeong Hun Kim, Kihwang Lee

**Affiliations:** 1 Department of Ophthalmology, Ajou University School of Medicine, 164 World Cup-ro, Yeongtong-gu, Suwon, 16499 South Korea; 2 Department of Cardiology, Chuncheon Sacred Heart Hospital, Hallym University College of Medicine, Chuncheon, South Korea; 3 Department of Chemistry, Mount Holyoke College, South Hadley, MA USA; 4 Department of Cardiology, Ajou University School of Medicine, Suwon, South Korea; 5 Fight against Angiogenesis-Related Blindness (FARB) Laboratory, Clinical Research Institute, Seoul National University Hospital, Seoul, South Korea; 6 Department of Biomedical Sciences, Seoul National University College of Medicine, Seoul, South Korea; 7 Department of Ophthalmology, Seoul National University College of Medicine, 101 Daehak-ro, Jongno-gu, Seoul, 03080 South Korea

**Keywords:** Cardiomyopathy, Diabetic retinopathy, Diastolic dysfunction, Microcirculation

## Abstract

**Background:**

To investigate the association between diabetic retinopathy (DR) and myocardial dysfunction in patients with type 2 diabetes and dilated cardiomyopathy (dCMP).

**Methods:**

Data were collected retrospectively from 89 patients with dCMP (46 with type 2 diabetes and 43 without diabetes) and no evidence of coronary artery disease. Echocardiographic parameters and laboratory data, including lipid profiles and fundus findings, were obtained from medical records. A left ventricular ejection fraction (LVEF) less than 40% was considered impaired systolic function, while an E/E′ ratio greater than 15 was considered elevated left ventricular (LV) filling pressure.

**Results:**

Baseline characteristics show that LVEF was not significantly different between patients with and without diabetes or between diabetic patients with and without DR. Among the diastolic function parameters, patients with DR exhibited higher E/E′ ratios (left ventricular filling pressures) than patients without DR (23.75 ± 13.37 vs 11.71 ± 3.50, *P* = 0.022). Logistic regression analysis revealed that statin use lowered the risk of impaired systolic dysfunction in all patients (odds ratio (OR) 0.33, 95% confidence interval (CI) 0.12–0.92, *P* = 0.034) and in patients with diabetes (OR 0.273, 95% CI 0.08–0.99, *P* = 0.049), while the presence of DR was associated with a higher risk of elevated LV filling filling pressure in patients with diabetes (OR 18.00, 95% CI 1.50–216.62, *P* = 0.023).

**Conclusions:**

In conclusion, DR was associated with elevated LV filling pressure in patients with dCMP. DR may not only represent microvascular long-term complications in patients with diabetes but may also be associated with more advanced form of diastolic dysfunction among diabetic patients with cardiomyopathy.

**Electronic supplementary material:**

The online version of this article (doi:10.1186/s12933-017-0566-y) contains supplementary material, which is available to authorized users.

## Background

Cardiovascular disease (CVD), including coronary artery disease and stroke, is the leading cause of death in patients with diabetes and directly related to atherosclerosis [1, 2]. Although the excessive risk of CVD in patients with diabetes may be due to common comorbidities, such as dyslipidemia, hypertension, and smoking, it is well known that diabetes alone can induce molecular changes in the heart [2]. The “common soil” hypothesis of diabetic complications has been introduced through several studies on the molecular mechanisms of diabetes [1–3]. Chronic hyperglycemia results in many microvascular complications in the eyes, nerves and kidneys, as well as higher risk of all macrovascular complications, including coronary and cerebrovascular disease [1, 4]. Diabetic retinopathy (DR), one of the major microvascular complications of diabetes, is also known to predict cardiovascular diseases and CVD-related death in individuals with type 2 diabetes [5].

Dilated cardiomyopathy (dCMP) is defined by the presence of left ventricular systolic dysfunction in the absence of an abnormal loading condition or significant coronary artery disease [6]. Endocrine disorders, including diabetes, are known to be associated with dCMP. Recent studies have revealed the presence of diabetic cardiomyopathy, a rare condition of myocardial dysfunction without coronary artery disease [1, 2, 7]. This term was first introduced by Rubler et al. [8] in 1972 and described patients with diabetes and congestive heart failure with normal coronary arteries. The exact pathophysiological mechanisms are still under investigation, while oxidative stress, impaired mitochondrial function, activation of the renin-angiotensin system, and altered substrate metabolism have been suggested as possible contributors to the pathogenesis [2, 7]. Since these mechanisms share common pathways with diabetic microvascular complications, diabetic cardiomyopathy is considered to indicate a microvascular component [1].

The presence of DR in diabetic patients suggests that microvascular complications are manifested clinically. Accordingly, we performed a retrospective clinical study to investigate the association of diabetes or DR with myocardial function in patients with type 2 diabetes with non-ischemic dCMP compared with that in patients without diabetes.

## Methods

The medical records of patients diagnosed with cardiomyopathy between 1994 and 2015 and followed by the Ophthalmology and Cardiology departments of Ajou University Hospital (Suwon, Korea) were retrospectively reviewed. This study complied with the Declaration of Helsinki and was approved by the Institutional Review Board of Ajou University Hospital (#AJIRB-MED-MDB-16-542). Patients were excluded if fundus examinations revealed any retinal vascular diseases other than diabetic retinopathy; without records of fundus findings and echocardiographic results; or with myocardial dysfunction related to secondary causes. Detailed exclusion criteria were as follows: the presence of significant coronary artery disease (>50% stenosis of at least one major coronary artery) confirmed by coronary angiography or non-performed angiography, or myocardial dysfunction resulted from significant valvular disease (symptomatic patients or asymptomatic patients with criteria of valvular heart disease by 2014 AHA/ACC guidelines [9]) or abnormal hemodynamic loading conditions. We also excluded patients with hypertrophic and restrictive cardiomyopathy to avoid heterogeneity in the echocardiographic data.

Demographic and clinical factors were obtained from medical records: age, gender, body mass index, general medical illness, presence of diabetes and DR, serum lipid profile, estimated glomerular filtration rate (eGFR), and medications including anticoagulants, β-blockers, angiotensin-converting enzyme inhibitor (ACEI), angiotensin receptor blocker (ARB), calcium channel blocker (CCB), diuretics and statins. Those without diabetes were defined by HbA1c <6.5%, fasting blood glucose <126 mg/dL, and lack of antidiabetic agents, verified from medical records. Fundus findings and echocardiographic parameters were also obtained from medical records. The collected echocardiographic data included left ventricular ejection fraction (LVEF), left ventricular fractional shortening (LVFS), left ventricular end-diastolic dimension (LVEDD), left ventricular end-systolic dimension (LVESD), left ventricular mass index (LVMI), posterior wall thickness at end-diastole (PWd) and at end-systole (PWs), relative wall thickness (RWT), peak early diastolic mitral inflow velocity (E), and peak late diastolic mitral inflow velocity (A). The early diastolic mitral annular velocity (E′) was averaged from the two measurements at septal and lateral sides of mitral annulus. The presence and severity of DR were obtained from medical records of ophthalmology department, based on the traditional Early Treatment Diabetic Retinopathy Study (ETDRS) grading system using fundus photos and fluorescence angiography of patients by an experienced retina specialist.

Statistical analysis was performed using SPSS software (version 23.0, SPSS, Chicago, IL). Shapiro–Wilk test was used for the assessment of the Gaussian distribution, and nonparametric tests were applied if the assumption of normality was violated. Categorical variables were compared using the Chi square test, and continuous variables were compared using independent *t* test, Mann–Whitney test, or Kruskal–Wallis test. Logistic regression analysis was performed to evaluate factors associated with impaired systolic function or elevated left ventricular (LV) filling pressure. Systolic dysfunction was defined as LVEF <40%, while elevated LV filling pressure was defined as E/E′ ratio >15 [10–14]. *P* < 0.05 were considered statistically significant.

## Results

89 patients with dCMP were included in this study, and their demographic characteristics are summarized in Table 1. In the laboratory profile, creatinine was increased in patients with diabetes, and eGFR was decreased in these patients. Hypertension was more common in patients with diabetes, while the use of statins was also more frequent in patients with diabetes. A subgroup analysis of the patients with diabetes was also performed, and 29 patients had DR, and 17 did not have DR (Table 2). Among the patients with DR, 18 presented with non-proliferative DR, and 11 presented with proliferative DR. The creatinine levels were significantly higher in the patients with DR. Total cholesterol and the levels of high-density lipoprotein cholesterol were higher in patients without DR.

**Table 1:** Baseline characteristics of patients with dilated cardiomyopathy with or without diabetes

Variable	DM group	No DM group	*P* value
No. of patients	46	43	
Age (years)	60.9 ± 11.2	61.6 ± 13.2	0.758
Male, N (%)	29 (63%)	24 (56%)	0.487
Body mass index (kg/m^2^)	24.0 ± 3.2	23.8 ± 4.0	0.792
Hypertension, N (%)	28 (61%)	9 (21%)	<0.001*
Laboratory profiles			
Creatinine (mg/dL)	2.4 ± 2.8	1.1 ± 0.2	0.006^†^
eGFR (mL/min/1.73 m^2^)	53.1 ± 31.2	70.6 ± 17.0	0.002^†^
Total cholesterol (mg/dL)	167.6 ± 39.2	159.6 ± 40.1	0.361
Triglycerides (mg/dL)	130.3 ± 78.2	140.5 ± 121.9	0.874
HDL cholesterol (mg/dL)	45.0 ± 14.3	47.3 ± 12.9	0.386
LDL cholesterol (mg/dL)	92.7 ± 37.0	97.3 ± 23.6	0.600
Medication, N (%)			
Anticoagulants	32 (70%)	31 (72%)	0.793
β-Blockers	26 (57%)	23 (53%)	0.774
ACEI/ARB	40 (87%)	38 (88%)	0.839
CCB	1 (2%)	2 (5%)	0.518
Diuretics	28 (61%)	29 (67%)	0.518
Statins	15 (33%)	6 (14%)	0.038*

*ACEI* angiotensin-converting enzyme inhibitor, *ARB* angiotensin receptor blocker, *CCB* calcium channel blocker, *DM* diabetes mellitus, *eGFR* estimated glomerular filtration rate, *HDL* high-density lipoprotein, *LDL* low-density lipoprotein

* *P* < 0.05 by Chi square test

^†^ *P* < 0.05 by independent *t* test or Mann–Whitney test

**Table 2:** Baseline characteristics of patients with diabetes and dilated cardiomyopathy with or without retinopathy

Variable	DR group	No DR group	*P* value
No. of patients	29	17	
Age (years)	60.4 ± 9.3	61.8 ± 14.1	0.487
Male, N (%)	17 (59%)	12 (71%)	0.417
Body mass index (kg/m^2^)	23.5 ± 3.2	25.0 ± 3.0	0.136
Hypertension, N (%)	19 (66%)	9 (53%)	0.399
Laboratory profiles			
HbA1c (%)	7.9 ± 1.8	7.4 ± 2.1	0.230
Creatinine (mg/dL)	3.1 ± 3.3	1.1 ± 0.5	0.007*
eGFR (mL/min/1.73 m^2^)	43.1 ± 29.8	71.9 ± 25.1	0.003*
Total cholesterol (mg/dL)	157.3 ± 36.2	190.8 ± 36.6	0.009*
Triglycerides (mg/dL)	112.9 ± 55.1	163.6 ± 105.1	0.284
HDL cholesterol (mg/dL)	41.7 ± 12.2	50.9 ± 16.5	0.049*
LDL cholesterol (mg/dL)	88.7 ± 37.6	100.6 ± 36.8	0.468
Medication			
Anticoagulants	18 (62%)	14 (82%)	0.149
β-Blockers	18 (62%)	8 (47%)	0.322
ACEI/ARB	25 (86%)	15 (88%)	0.844
CCB	1 (3%)	0 (0%)	0.439
Diuretics	19 (66%)	9 (53%)	0.399
Statins	11 (38%)	4 (24%)	0.315
DPP4 inhibitors	5 (17%)	1 (6%)	0.270
Metformin	7 (24%)	3 (18%)	0.606
Sulfonylurea	10 (34%)	6 (35%)	0.956

*ACEI* angiotensin-converting enzyme inhibitor, *ARB* angiotensin receptor blocker, *CCB* calcium channel blocker, *DPP4* dipeptidyl peptidase 4, *DR* diabetic retinopathy, *eGFR* estimated glomerular filtration rate, *HDL* high-density lipoprotein, *LDL* low-density lipoprotein

* *P* < 0.05 by independent *t* test or Mann–Whitney test

The echocardiographic parameters are described in Table 3 (patients with or without diabetes) and Table 4 (patients with diabetes and with or without DR). There were no significant differences in the parameters between the patients with and without diabetes. However, the values of the E′ and E/E′ ratio among the diastolic parameters showed significant differences according to the presence of DR (*P* = 0.021 and *P* = 0.022, respectively). These parameters were also significantly different when compared among those without diabetes, those with diabetes but no DR, and those with DR (*P* = 0.019 for E′ and *P* = 0.022 for E/E′ ratio, respectively) (Additional file 1: Tables S1, S2).

**Table 3:** Echocardiographic parameters of patients with dilated cardiomyopathy with or without diabetes

Variable	DM group	No DM group	*P* value*
LVEDD (mm)	58.0 ± 7.9	58.7 ± 10.1	0.967
LVESD (mm)	46.5 ± 11.1	45.4 ± 11.8	0.630
IVSd (mm)	10.4 ± 2.0	9.7 ± 1.6	0.122
IVSs (mm)	13.2 ± 2.9	12.7 ± 2.7	0.381
PWd (mm)	10.5 ± 2.0	9.7 ± 1.6	0.077
PWs (mm)	13.9 ± 2.4	13.6 ± 2.4	0.504
RWT	0.4 ± 0.1	0.3 ± 0.1	0.277
LVEF (%)	37.3 ± 16.9	39.5 ± 16.8	0.546
LVFS (%)	20.9 ± 9.1	22.7 ± 9.6	0.402
LV mass (g)	253.0 ± 78.7	229.0 ± 77.5	0.178
LV mass index (g/m^2^)	149.5 ± 41.0	141.5 ± 45.4	0.391
E (m/s)	0.80 ± 0.30	0.76 ± 0.26	0.796
A (m/s)	0.76 ± 0.27	0.69 ± 0.18	0.385
E/A ratio	1.31 ± 0.96	1.13 ± 0.78	0.701
E′ (m/s)	0.05 ± 0.02	0.05 ± 0.02	0.095
E/E′ ratio	19.32 ± 12.20	14.85 ± 5.81	0.516

*A* peak late diastolic mitral inflow velocity, *E* peak early diastolic mitral inflow velocity, *E′* early diastolic mitral annular velocity, *IVSd* interventricular septal thickness at end-diastole, *IVSs* interventricular septal thickness at end-systole, *LV* left ventricular, *LVDd* left ventricle dimension at end-diastole, *LVDs* left ventricle dimension at end-systole, *PWd* posterior wall thickness at end-diastole, *PWs* posterior wall thickness at end-systole, *RWT* relative wall thickness

* *P* < 0.05 by independent *t*-test or Mann–Whitney test

**Table 4:** Echocardiographic parameters of patients with diabetes and dilated cardiomyopathy with or without retinopathy

Variable	DR group	No DR group	*P* value
LVDd (mm)	58.1 ± 6.2	57.7 ± 10.3	0.569
LVDs (mm)	46.2 ± 8.9	47.1 ± 14.3	0.833
IVSd (mm)	10.6 ± 2.2	10.2 ± 1.6	0.843
IVSs (mm)	13.2 ± 3.2	13.2 ± 2.1	0.900
PWd (mm)	10.6 ± 2.2	10.4 ± 1.7	0.852
PWs (mm)	13.9 ± 2.6	13.9 ± 2.1	0.908
RWT	0.4 ± 0.1	0.4 ± 0.1	0.726
LVEF (%)	35.4 ± 16.0	40.7 ± 18.3	0.312
LVFS (%)	19.5 ± 8.5	23.8 ± 10.1	0.191
LV mass (g)	253.4 ± 74.4	252.2 ± 88.8	0.964
LV mass index (g/m^2^)	154.9 ± 38.6	140.2 ± 44.3	0.438
E (m/s)	0.89 ± 0.32	0.67 ± 0.21	0.088
A (m/s)	0.74 ± 0.31	0.80 ± 0.20	0.619
E/A ratio	1.55 ± 1.08	0.78 ± 0.16	0.231
E′ (m/s)	0.04 ± 0.02	0.06 ± 0.01	0.021*
E/E′ ratio	23.75 ± 13.37	11.71 ± 3.50	0.022*

*A* peak late diastolic mitral inflow velocity, *E* peak early diastolic mitral inflow velocity, *E′* early diastolic mitral annular velocity, *IVSd* interventricular septal thickness at end-diastole, *IVSs* interventricular septal thickness at end-systole, *LV* left ventricular, *LVDd* left ventricle dimension at end-diastole, *LVDs* left ventricle dimension at end-systole, *PWd* posterior wall thickness at end-diastole, *PWs* posterior wall thickness at end-systole, *RWT* relative wall thickness

* *P* < 0.05 by independent *t* test or Mann–Whitney test

Logistic regression analysis was performed to investigate the risk factors associated with impaired systolic or diastolic dysfunction in patients with dCMP for the following factors: age, gender, hypertension, diabetes, DR, eGFR, lipid profiles, and systemic medications. Among these factors, the regression analysis investigating the risk factors associated with systolic dysfunction showed that statin use significantly lowered the degree of impairment in LVEF in the whole study population (OR 0.33, 95% CI 0.12–0.92, *P* = 0.034, Table 5) and patients with diabetes (OR 0.273, 95% CI 0.08–0.99, *P* = 0.049, Table 6). For the factors associated with diastolic dysfunction, the presence of DR was significantly associated with the risk of an E/E′ ratio >15 in the patients with diabetes (OR 18.00, 95% CI 1.50–216.62, *P* = 0.023, Table 6), while the other factors were not significant.

**Table 5:** Logistic regression analysis between patients with impaired myocardial function and those with preserved myocardial function

Variable	Systolic dysfunction		Diastolic dysfunction	
	OR (95% CI)	*P* value	OR (95% CI)	*P* value
Age	0.984 (0.951–1.020)	0.381	1.005 (0.957–1.054)	0.855
Gender	0.767 (0.328–1.793)	0.540	1.800 (0.522–6.204)	0.352
Diabetes	1.789 (0.770–4.154)	0.176	1.852 (0.545–6.287)	0.323
Hypertension	1.008 (0.433–2.349)	0.985	2.857 (0.822–9.930)	0.099
eGFR	0.986 (0.969–1.003)	0.116	0.969 (0.941–0.998)	0.039*
Statin	0.329 (0.118–0.922)	0.034*	0.648 (0.158–2.656)	0.546

*CI* confidence interval, *eGFR* estimated glomerular filtration rate, *OR* odd ratio

* *P* < 0.05 by logistic regression analysis

**Table 6:** Logistic regression analysis between patients with diabetes and impaired myocardial function and those with preserved myocardial function

Variable	Systolic dysfunction		Diastolic dysfunction	
	OR (95% CI)	*P* value	OR (95% CI)	*P* value
Age	0.993 (0.941–1.048)	0.794	1.001 (0.923–1.086)	0.981
Gender	1.950 (0.545–6.974)	0.304	8.000 (0.711–90.001)	0.092
DR	2.500 (0.725–8.598)	0.146	18.000 (1.496–216.620)	0.023*
Hypertension	0.983 (0.292–3.310)	0.979	4.500 (0.374–54.155)	0.236
eGFR	0.983 (0.962–1.004)	0.120	0.963 (0.925–1.002)	0.066
Statin	0.273 (0.075–0.992)	0.049*	1.333 (0.204–8.708)	0.764

*CI* confidence interval, *DR* diabetic retinopathy, *eGFR* estimated glomerular filtration rate, *OR* odd ratio

* *P* < 0.05 by logistic regression analysis

## Discussion

### Microvascular complication (DR) and macrovascular complication (CVD) of diabetes

Diabetes is responsible for various cardiovascular complications, such as myocardial infarction, stroke, and peripheral vascular disease [7]. These diseases are at least twofold more common in patients with type 2 diabetes than in individuals without diabetes [4]. CVD is the most important complication in diabetic people, and coronary artery disease is the main cause of death in over 50% of patients with type 2 diabetes [15]. Traditionally, microvascular and macrovascular complications were studied and treated as distinct aspects of diabetes, while many evidences suggested common pathophysiological features between these diabetic complications [16]. However, a close relationship between microvascular complication (DR) and macrovascular complication (CVD) recently. DR in patients with normal renal function and without cardiovascular disease was associated with a higher atherosclerotic burden in the carotid arteries [16]. The presence of DR was independently associated with diastolic and systolic impaired function, both at rest and stress, evaluated by global longitudinal strain and diastolic function reserve index, and might be a useful predictor of major adverse cardiac events such as cardiac death, myocardial infarction, and acute heart failure following percutaneous coronary intervention [21, 22]. The progression of DR, the presence of proliferative DR (PDR), was associated with higher risk of having coronary heart disease (CHD), and was correlated with the severity of CHD [20]. In similar contexts, the impairment of the heart muscle perfusion at stress and rest in PDR patients was more frequent than in the non-proliferative DR (NPDR) patients and diabetic patients without DR suggesting PDR as a useful indicator of heart muscle perfusion disturbance in SPECT studies [18].

### Microvascular complication (DR) and cardiac microangiopathy (diabetic cardiomyopathy)

In addition to these macrovascular complications, there is evidence of microvascular damage in the hearts of patients with diabetes [1, 7]. The disease entity of “diabetic cardiomyopathy” has been introduced and is characterized by the presence of myocardial dysfunction with non-significant coronary arteries [2, 7, 8]. The exact pathophysiology of diabetic cardiomyopathy is unknown, but metabolic changes such as hyperglycemia, dyslipidemia, insulin resistance, and activation of the renin-angiotensin system are thought to result in myocardial fibrosis [1, 2]. Structural changes such as endothelial swelling and/or degeneration and thickening of the capillary basement membrane are also present in diabetic cardiomyopathy, which suggests a similar pathogenesis with microangiopathy [1, 23]. Based on these findings, the clinical phenotype of diabetic cardiomyopathy mostly corresponded to dCMP characterized by left ventricular dilatation and left ventricular systolic dysfunction [2, 6, 24].

The presence of microangiopathy in the heart was reported to show thickening of the capillary basement membrane, microvascular spasm, and capillary microaneurysms [25]. These are also representative features of DR, which suggests a common pathophysiology in the heart. The metabolic complications of diabetes originate from hyperglycemia, which results in the formation of advanced glycation end products and production of reactive oxygen species, followed by vascular endothelial dysfunction [25–27]. Moreover, recent study proposed that hyperglycemia-related hyperosmolarity promoted inflammation and angiogenesis by COX-2 expression, and may have a role not only in microvascular disease but also in macrovascular disease [17]. These factors act alone or in combination to promote myocardial fibrosis due to diabetes-induced microangiopathy [25–27].

### DR and diastolic dysfunction in dCMP

Our study focused on the association of diabetes or DR with dCMP, and we especially investigated the detailed changes in echocardiographic findings. The systolic and diastolic parameters revealed no significant difference according to the presence of diabetes because the study population was confined to those diagnosed with dCMP. However, the parameters, such as E/A ratio, E′ or E/E′ ratio, that represent diastolic dysfunction, were impaired in patients with DR compared to those in patients with diabetes and without DR, which suggests that DR rather than diabetes may be associated with worsened form of diastolic dysfunction in dCMP [7, 28, 29]. It is believed that the use of ACEI/ARB for patients with diabetes as the first choice of antihypertensive drugs in general may protect against the fibrotic changes in the left ventricle, since increased activation of the renin-angiotensin-aldosterone pathway leads to fibrosis formation [28]. It is only after the small vessel diseases, such as retinopathy or nephropathy, are present, which indicate widespread systemic microcirculation diseases, that the characteristics of diabetic cardiomyopathy are prominently evident.

**Fig. 1:**
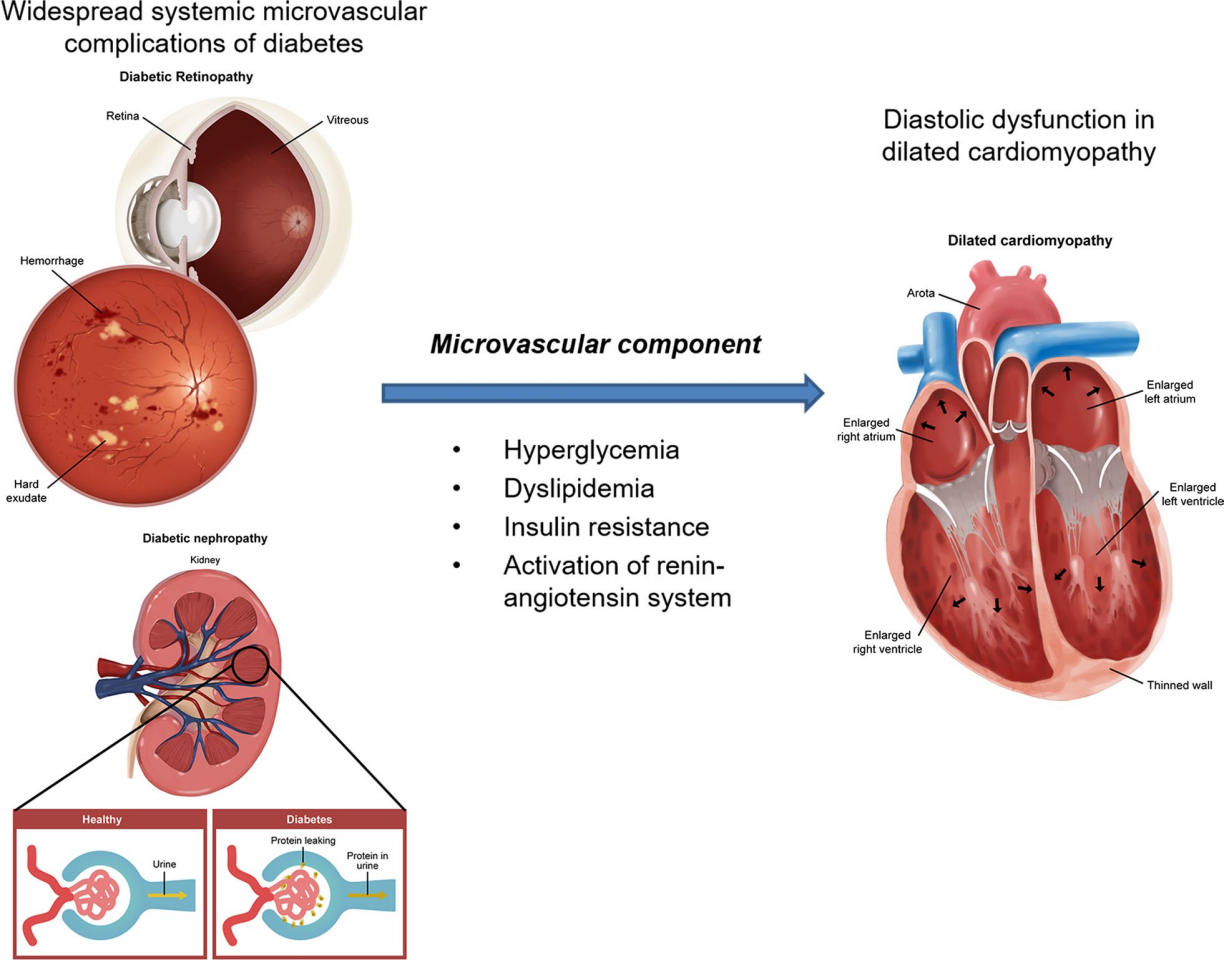
Pathophysiological mechanisms that lead to diastolic dysfunction in dilated cardiomyopathy

The presence of DR suggests that the patient is experiencing microvascular complications. DR, one of the major microvascular complications in patients with diabetes, has been investigated as a potential predictor for cardiovascular diseases [5, 19, 30, 31]. Studies performed with patients with type 2 diabetes have revealed that the presence of DR is associated with an excess risk of heart failure or cardiovascular mortality [5, 30]. Furthermore, decreased eGFR was noted in patients with diabetes compared to that in non-diabetic patients, as well as in patients with DR compared to patients with diabetes and without DR. The decreased eGFR shown in this study may suggest systemic microvascular damage along with DR, since the associations between diabetic nephropathy and retinopathy have been well documented [32]. These studies suggest a possible contribution of microvascular damage to macrovascular diseases in patients with diabetes [5, 30]. However, lower eGFR did not significantly increase the risk of diastolic dysfunction (OR 0.969, 95% CI 0.941–0.998, *P* = 0.039). Altogether, the presence of retinopathy rather than nephropathy could be used as a clinical feature to determine whether patients with diabetes are likely to have elevated LV filling pressure in dCMP (Fig. 1).

### Statin use and dCMP

Among the systemic medications, statin use was associated with a lower degree of systolic dysfunction in patients with dCMP. There are several experimental studies that have reported the protective effect of statin use on diabetic cardiomyopathy [33, 34], while controversy exists in human studies [35, 36]. We previously demonstrated the protective effect of statin use in DR [37], which may also be protective in diabetes-induced cardiac microangiopathy as shown in this study. However, further larger scale studies are needed to verify the protective effect of statin on dCMP, as the small number of patients included in this study is a major limitation.

### Limitations

The present study has also other limitations with regard to the retrospective design; more detailed assessment of diastolic profiles such as pulmonary venous flow, left atrial volume, or tricuspid regurgitation pressure gradient was not available. Furthermore , the measurement of LV end-diastolic pressure by catheter examination would be informative, which was not available in this retrospective study. Cohort studies with larger numbers of subjects may be needed for further investigation of diabetes, DR, and cardiomyopathy. Some large ranges of 95% CI in the logistic regression analyses, which were especially common in the subgroup of patients with diabetes, may be narrowed with statistical significance if analyzed with a larger number of patients.

## Conclusions

In conclusion, DR was associated with diastolic dysfunction in patients with dCMP. DR may not only represent microvascular long-term complications in patients with diabetes but may also be associated with more advanced form of diastolic dysfunction among diabetic patients with cardiomyopathy. Based on our findings, dCMP patients with DR should be encouraged to increase the frequency of cardiovascular follow-up, and intensive health education on life style modification and special attention to medication adherence are needed to avoid worsening the condition.

## Additional file

**Additional file 1: Table S1.** Echocardiographic parameters of patients with dilated cardiomyopathy with or without diabetes and diabetic retinopathy. **Table S2.** Echocardiographic parameters of patients with dilated cardiomyopathy and diabetic retinopathy.

## Abbreviations

A: peak late diastolic mitral inflow velocity
ACEI: angiotensin-converting enzyme inhibitor
ARB: angiotensin receptor blocker
CCB: calcium channel blocker
CI: confidence interval
CVD: cardiovascular disease
dCMP: dilated cardiomyopathy
DR: diabetic retinopathy
E: peak early diastolic mitral inflow velocity
E′: early diastolic mitral annular velocity
eGFR: estimated glomerular filtration rate
LV: left ventricular
LVEDD: left ventricular end-diastolic dimension
LVESD: left ventricular end-systolic dimension
LVEF: left ventricular ejection fraction
LVFS: left ventricular fractional shortening
LVMI: left ventricular mass index
OR: odd ratio
PWd: posterior wall thickness at end-diastole
PWs: posterior wall thickness at end-systole
RWT: relative wall thickness
