# Supplementary material for: Diabetic retinopathy is associated with diastolic dysfunction in type 2 diabetic patients with non-ischemic dilated cardiomyopathy
Source: Cardiovasc Diabetol. 2017 Jul 6;16:82. doi: 10.1186/s12933-017-0566-y (PMC5500965; doi:10.1186/s12933-017-0566-y)
Supplement: Supplementary file 1 — Additional file 1: Table S1. Echocardiographic parameters of patients with dilated cardiomyopathy with or without diabetes and diabetic retinopathy. Table S2. Echocardiographic parameters of patients with dilated cardiomyopathy and diabetic retinopathy. [file 12933_2017_566_MOESM1_ESM.docx]

**Table S1.** Echocardiographic parameters of patients with dilated cardiomyopathy with or without diabetes and diabetic retinopathy

| **Variable** | **DR group** | **DM & No DR group** | **No DM group** | ***P* value** |
| --- | --- | --- | --- | --- |
| E/A ratio | 1.55 ± 1.08 | 0.78 ± 0.16 | 1.13 ± 0.78 | 0.573 |
| E’ (m/sec) | 0.04 ± 0.02 | 0.06 ± 0.01 | 0.05 ± 0.02 | 0.019^*^ |
| E/E’ ratio | 23.75 ± 13.37 | 11.72 ± 3.50 | 14.85 ± 5.81 | 0.022^*^ |

A, peak late diastolic mitral inflow velocity; E, peak early diastolic mitral inflow velocity; E’, early diastolic mitral annular velocity. ^*^*P* value < 0.05 by Kruskal-Wallis test.

**Table S2.** Echocardiographic parameters of patients with dilated cardiomyopathy and diabetic retinopathy

| **Variable** | **NPDR (N = 18)** | **PDR (N = 11)** | ***P* value** |
| --- | --- | --- | --- |
| E/A ratio | 1.49 ± 1.26 | 1.70 ± 0.52 | 0.411 |
| E’ (m/sec) | 0.03 ± 0.01 | 0.07 ± 0.02 | 0.024^*^ |
| E/E’ ratio | 26.56 ± 14.28 | 15.33 ± 5.03 | 0.227 |

A, peak late diastolic mitral inflow velocity; E, peak early diastolic mitral inflow velocity; E’, early diastolic mitral annular velocity; NPDR, non-proliferative diabetic retinopathy; PDR, proliferative diabetic retinopathy. ^*^*P* value < 0.05 by Mann-Whitney test.
